# Dual Response Site Fluorescent Probe for Highly Sensitive Detection of Cys/Hcy and GSH In Vivo through Two Different Emission Channels

**DOI:** 10.3390/bios12111056

**Published:** 2022-11-21

**Authors:** Huiling Hou, Qi Liu, Xiangbao Liu, Shuang Fu, Hongguang Zhang, Shuang Li, Song Chen, Peng Hou

**Affiliations:** 1 Achievement Transformation Center, Qiqihar Medical University, Qiqihar 161006, China; 2 Research Institute of Medicine & Pharmacy, Qiqihar Medical University, Qiqihar 161006, China; 3 College of Pharmacy, Qiqihar Medical University, Qiqihar 161006, China

**Keywords:** biothiols, dual-site, coumarin, fluorescent probe

## Abstract

Much research has demonstrated that metabolic imbalances of biothiols are closely associated with the emergence of different types of disease. In view of the significant effect of biothiols, quantitative evaluation and discrimination of intracellular Cys/Hcy and GSH in complex biological environments is very important. In this study, probe **CDS-NBD**, synthesized by attaching 2,4-dinitrobenzenesulfonate (DNBS, site 1) and nitrobenzoxadiazole (NBD, site 2) as the highly sensitive and selective dual response site for thiols onto the coumarin derivative 7-hydroxycoumarin-4-acetic acid, exhibited large separation of the emission wavelengths, fast response, notable fluorescence enhancement, excellent sensitivity and selectivity to Cys/Hcy and GSH over other biological species. Additionally, **CDS-NBD** could make a distinction between two different fluorescent signals, GSH (an obvious blue fluorescence) and Cys/Hcy (a mixed blue-green fluorescence). Further study on imaging of Cys/Hcy and GSH in vivo by employing probe **CDS-NBD** could also be successfully achieved.

## 1. Introduction

Cysteine (Cys)/homocysteine (Hcy) and glutathione (GSH), as vital low-weight bio-molecules containing a sulfhydryl group (-SH), play vital and irreplaceable roles in maintaining the appropriate redox status in diverse physiological processes [1,2,3]. Cys serves as a critical substrate for the synthesis of GSH and acetyl coenzyme A and participates in amino acid transport and detoxification of heavy metal poisoning in biological systems. Hcy is a methylene homolog of Cys, involved in constructing DNA and methylation catalyzed to produce Cys in organisms. As the most abundant of biothiols, the level of GSH is about 1–10 mmol/L in cells. It maintains the redox balance of cellular homeostasis against oxidative stress [4,5,6,7]. During recent years, much research has demonstrated that metabolic imbalances of biothiols are closely associated with the emergence of different types of disease [8,9,10]. The loss of Cys can cause growth retardation, metabolic disorders, type 2 diabetes, and neurodegenerative disorders. The elevated plasma of Hcy concentration in the serum is regarded as a root hazard element for cardiovascular disease and cognitive impairment in the elderly. Abnormal fluctuation of GSH is tightly relative to atherosclerosis, cancer, and Alzheimer’s disease. In view of the significant effect of biothiols, quantitative evaluation and discrimination of intracellular Cys/Hcy and GSH in complex biological environments is very important.

In the last decade, given the apparent advantages of low cost, rapid response, excellent sensitivity, non-invasiveness, high resolution, and ease of visualization, fluorescent probes have drawn considerable attention in the analysis of thiols [11,12,13,14,15,16,17,18,19,20]. However, most reported thiols probes give the same response toward Cys, Hcy, and GSH owing to their similar structures and properties. Moreover, although numerous selectivity-thiols probes employing only one reactive site could sense thiols via a single fluorescent channel, dual-site fluorescent probes possess many obvious advantages [21,22,23,24,25,26], such as desirable selectivity, improved sensitivity, rapid response time, and minimized background. 

**Scheme 1:**
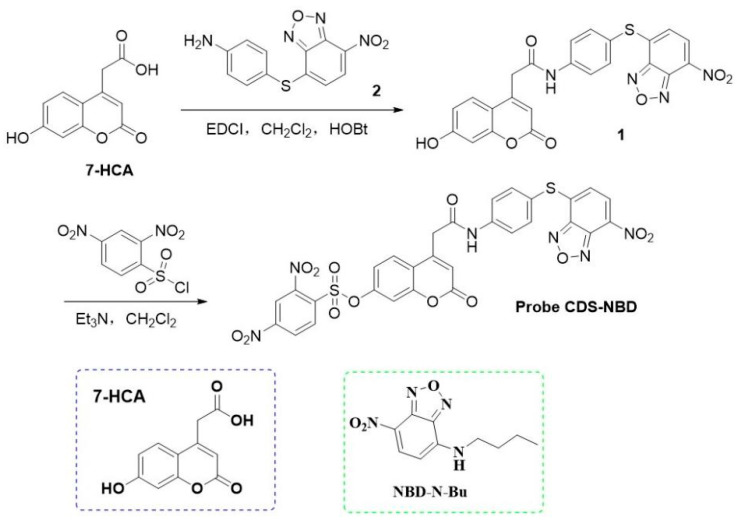
The synthesis route for probe **CDS-NBD**.

**Scheme 2:**
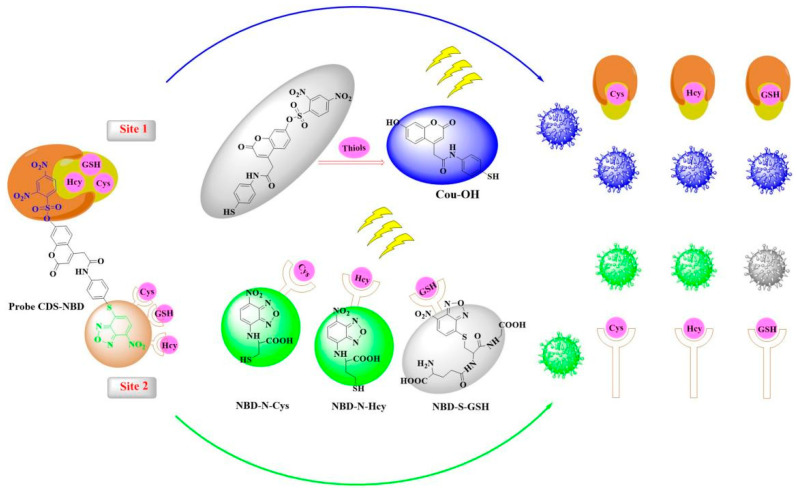
The design strategy and proposed sensing mechanism of **CDS-NBD** toward biothiols.

Due to the above considerations, by using 2,4-dinitrobenzenesulfonate (DNBS, site 1) and nitrobenzoxadiazole (NBD, site 2) as the highly sensitive and selective dual response site for thiols, a novel fluorescent probe **CDS-NBD** based on coumarin derivative 7-hydroxycoumarin-4-acetic Acid (**7-HCA**) for the detection of Cys/Hcy and GSH through two different emission channels is presented (Scheme 1). Probe **CDS-NBD** was a non-fluorescent compound due to the PET process arising from the dual quenching site (2,4-dinitrobenzenesulfonate and NBD moiety). Cys/Hcy could react with site 1 and site 2 in probe **CDS-NBD** to generate the blue fluorescent substance **Cou-OH** and the green fluorescent substance **NBD-N-Cys** or **NBD-N-Hcy**. However, GSH could not undergo the Smile rearrangement, so the blue fluorescent substance **Cou-OH** and the non-fluorescent substance **NBD-S-GSH** were obtained. Thus, probe **CDS-NBD** could make a distinction between GSH (an obvious blue fluorescence) and Cys/Hcy (a mixed blue-green fluorescence) by two different fluorescent signals (Scheme 2). In addition, dual-color fluorescence imaging experiments in living HeLa cells and zebrafish also demonstrated that **CDS-NBD** could serve as an effective tool for responding to Cys/Hcy and GSH. 

## 2. Results and Discussion

### 2.1. Design of Probe ***CDS-NBD***

Recently, single-molecule fluorescent probes with different reaction sites for simultaneously sensing a variety of related analytes in vitro and in vivo through two or more kinds of distinct fluorescence channels (Table S1) have attracted enormous attention [27,28,29]. In our design, 2,4-Dinitrobenzenesulfonate (site 1) [30] could serve as an excellent active site for identifying thiols (Cys, Hcy, and GSH) with high selectivity. Therefore, strong emission in the blue channel could be aroused after the reaction between site 1 in **CDS-NBD** and thiols. Moreover, the NBD group (site 2) [31] could easily react with Cys/Hcy to afford **NBD-N-Cys/Hcy**, enabling probe **CDS-NBD** to exhibit significant fluorescence changes at 557 nm. In contrast, no obvious green fluorescence signal was seen for GSH because the Smile rearrangement was forbidden. Furthermore, probe **CDS-NBD** adopted a dual-quenching strategy (site 1 and site 2), which could display the minimized background signal. For these reasons, we obtained a dual-site fluorescent probe **CDS-NBD** for simultaneously monitoring Cys/Hcy and GSH via two different emission channels. The HRMS, ^13^C NMR, and ^1^H NMR spectra of **CDS-NBD** are given in Figures S11–S16. 

### 2.2. Spectral Performances

**Figure 1:**
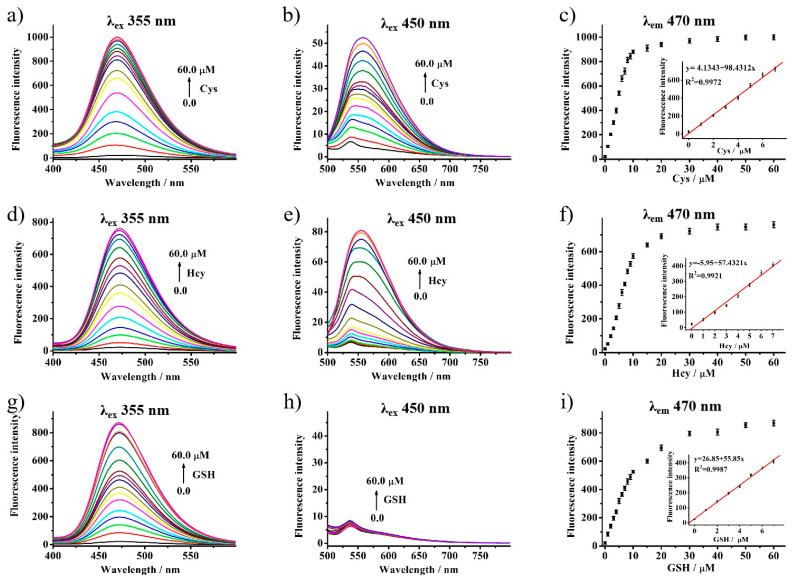
Fluorescence response of probe **CDS-NBD** (10.0 μM) with different biothiols (Cys, Hcy, GSH) in the 0.0–60.0 μM range, respectively. (**a**,**d**,**g**) λex = 355 nm, (**b**,**e**,**h**) λex = 450 nm, (**c**,**f**,**i**) The linear correlation between the concentration of biothiols (Cys, Hcy, GSH) and the emission intensity.

At first, we studied the fluorescence spectra of probe **CDS-NBD** (10.0 μM) by adding Cys/Hcy and GSH in PBS (20 mM, pH 7.4, containing 20% DMSO). **CDS-NBD** (10.0 μM) had essentially no signal both in the blue and green channel (Figure 1). However, when Cys/Hcy/GSH (0.0–60.0 μM) were added, a significant fluorescence signal at 470 nm (Figure 1a,d,g) under the 355 nm excited condition was observed, corresponding to that of **Cou-OH**. Importantly, fluorescence intensity was evidently increased (46-fold for Cys, 35-fold for Hcy, and 40-fold for GSH) after responding to the above three thiols (60.0 μM). As shown in Figure 1c,f,i, excellent linear relationships (R^2^ = 0.9972 for Cys, R^2^ = 0.9921 for Hcy, R^2^ = 0.9987 for GSH) with three thiols concentration in the range of 0.0–6.0 μM were obtained. In addition, the calculated limit of detection (LOD) of 10.0 μM probe **CDS-NBD** was calculated to be 32, 45, and 22 nM for Cys, Hcy, and GSH based on the fitting equations (Cys: y = 4.1343 +98.4312x, Hcy: 5.95 + 57.4321x, GSH: y = 26.85 + 55.85x). From Figure 1b,e, upon gradual addition of Cys/Hcy (0.0–60.0 μM), an intense fluorescence signal at 557 nm was found under the 450 nm excited condition, corresponding to that of **NBD-N-Cys/Hcy**. Additionally, the fluorescence intensity at 557 nm showed persistent and proportional enhancement along with the concentration of Cys/Hcy (0.0–60.0 μM). In stark contrast, **CDS-NBD** (10.0 μM) in response to GSH (60.0 μM) displayed little impact on the emission intensity at 557 nm (Figure 1h). These satisfactory sensing data of **CDS-NBD** for three thiols proved that **CDS-NBD** could report Cys/Hcy and GSH with desirable sensitivity through the blue and green emission channels. 

### 2.3. Selectivity Studies 

**Figure 2:**
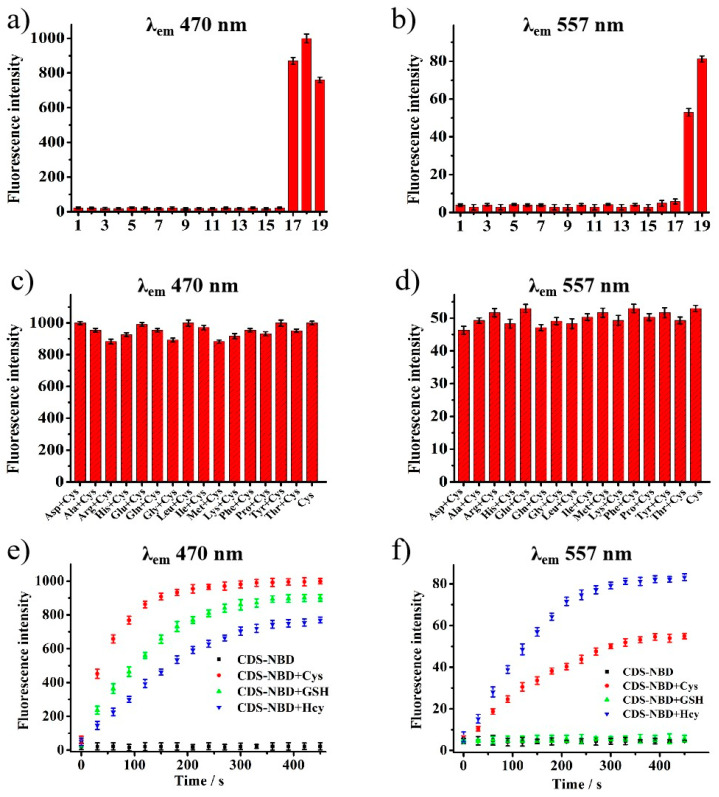
The fluorescence response of probe **CDS-NBD** (10.0 μM) toward various amino acids (1–16:100.00 μM for Asp, Ala, Arg, His, Glu, Gln, Gly, Leu, Ile, Met, Lys, Phe, Pro, Tyr, Thr and 17–19: 60.00 μM for GSH, Cys, Hcy) in PBS ((**a**): λem = 470 nm, (**b**): λem = 557 nm). The fluorescence response of probe **CDS-NBD** (10.0 μM) to Cys (60.0 μM) in the presence of other coexisting substances (100.00 μM) in PBS ((**c**): λem = 470 nm, (**d**): λem = 557 nm). The plot of fluorescence emission of probe **CDS-NBD** (10.0 μM) versus time with different biothiols (Cys, Hcy, GSH) at 470 nm (**e**) and at 577 nm (**f**).

To evaluate the specific sensing property of probe **CDS-NBD** towards biothiols, the selectivity of probe **CDS-NBD** (10.00 μM) was assessed by incubation with biologically related species. For emission at 470 nm (Figure 2a), Cys, Hcy, and GSH exhibited similar distinct changes of fluorescence enhancement, while obvious emission enhancement at 557 nm (Figure 2b) was detected by incubation of Cys/Hcy (60.00 μM) in comparison to that of GSH (60.00 μM). The results indicated that miscellaneous amino acids (including 100.00 μM for Asp, Ala, Arg, His, Glu, Gln, Gly, Leu, Ile, Met, Lys, Phe, Pro, Tyr, Thr) and biologically relevant ions (including 100.00 μM for K^+^, Zn^2+^, Mg^2+^, Na^+^, Ca^2+^, NO_3_^−^, CO_3_^2−^, NO_2_^−^, I^−^, SO_4_^2−^, SO_3_^2−^, F^−^, Cl^−^, Br^−^, ClO^−^) only induced very weak fluorescence variations over the whole scale of the experiment (Figures S4 and S5), probe **CDS-NBD** had a superior selectivity for Cys and Hcy at the dual-emission wavelength (470 nm and 557 nm), and high recognition activity for GSH only at 470 nm. As for the fluorescence responses of competitive analytes (such as Asp, Ala, Arg, His, Glu, Gln, Gly, Leu, Ile, Met, Lys, Phe, Pro, Tyr, Thr, K^+^, Zn^2+^, Mg^2+^, Na^+^, Ca^2+^, NO_3_^−^, CO_3_^2−^, NO_2_^−^, I^−^, SO_4_^2−^, SO_3_^2−^, F^−^, Cl^−^, Br^−^, ClO^−^), in Figure 2c,d and Figures S6 and S7, with the emission at 470 nm or 557 nm, all of the above-mentioned interfering substances triggered a negligible fluorescence change (F_c_) both at two emission wavelength with ranges of 1.01–0.89 (F_c_ = F_I_/F_r_, F_c_ was the ratio of fluorescence intensity change, F_I_ was the fluorescence intensity of probe **CDS-NBD** (10.00 μM) reacted with Cys (60.00 μM) + interfering substances (100.00 μM), F_r_ is the fluorescence intensity of probe **CDS-NBD** (10.00 μM) reacted with Cys (60.00 μM)). In other words, the probe **CDS-NBD** can serve as a monitoring tool for distinguishing and detecting corresponding biothiols (Cys/Hcy and GSH) over other biological species by different fluorescence signals. 

### 2.4. Response Time and pH Effect

In order to evaluate the real-time performance of a reactive fluorescent probe, the response dynamics of probe **CDS-NBD** with biothiols was conducted by testing the changes of fluorescence intensity with time under two different emissions at 470 nm and 557 nm. In fact, free probe **CDS-NBD** (10.00 μM) was non-emissive no matter how long it took owing to the dual quenching site in its structure. After the addition of three biothiols (60.00 μM) to the solution of probe **CDS-NBD** (10.00 μM) at 470 nm (Figure 2e), sharp increments of similar fluorescence intensities were observed. The reaction rate of Cys was slightly quicker than Hcy and GSH during the initial 100 s, and the response rate of probe **CDS-NBD** toward three biothiols was all speedy, which can rapidly maximize the fluorescence intensity in 350 s. It is notable that the fluorescence behavior of the solution at 557 nm emission wavelength (Figure 2f) was silent following GSH addition under the same conditions. In contrast, probe **CDS-NBD** showed fast fluorescence response to other biothiols (Cys and Hcy), which obtained saturation within 350 s. Therefore, 350 s was selected as the optimization time for the detection of biothiols in the following experiments.

As a significant influence factor in application environments, the effect of pH on the system was also investigated by examining the interactions of probe **CDS-NBD** toward Cys, Hcy, and GSH at different pH values from 2.0 to 12.0. As shown in Figures S8 and S9, in the absence of three biothiols, probe **CDS-NBD** (10.00 μM) was pH insensitive along with a wide pH range (from 2.0 to 11.0) at 470 nm and 557 nm. Upon addition of Cys and Hcy (60.00 μM), distinct enhancement of fluorescence signals at the emission of 470 nm and 557 nm were achieved within the pH 6.0–9.0 range, respectively. By contrast, the probe **CDS-NBD** (10.00 μM) only became sensitive at 470 nm toward GSH (60.00 μM) with pH values changed from 6.0 to 9.0, while the addition of GSH elicited inappreciable fluorescence changes at 557 nm between pH 2.0 and 12.0. Fortunately, the physiological environment (pH = 7.4) placed exactly in the excellent scope of probe detection toward biothiols, suggesting that probe **CDS-NBD** could be capable of sensing and differentiating biothiols in more complex environments.

### 2.5. Reaction Mechanism

To attest to the sensing mechanism of **CDS-NBD** toward thiols in this paper, we first used HRMS analyses to certify the reaction products between **CDS-NBD** and GSH/Cys. As we expected, **Cou-OH** [Cou-OH+Na]^+^ (cal. 350.0463) and **NBD-N-GSH** [**NBD-S-GSH**+Na]^+^ (cal. 493.0754) were the reaction products of **CDS-NBD** with GSH, characterized by the peaks (Figure S17) at 350.0498 and 493.0764. Similarly (Figure S18), **Cou-OH** [Cou-OH+Na]^+^ (cal. 350.0463) and **NBD-N-Cys**[**NBD-S-Cys**]^+^ (cal. 284.0215) were the reaction products of **CDS-NBD** with Cys. In addition, this mechanism was also investigated by spectral analyses. As is shown in Figures S1–S3, the control compound **7-HCA** and **Cou-OH** had a similar fluorescence spectrum. Meantime, the emission spectra of the control compounds **NBD-N-Bu** were quite similar to the emission spectra of **NBD-N-Cys**. These findings robustly proved the sensing mechanism of **CDS-NBD** toward thiols in Scheme 2. 

### 2.6. Cellular Imaging

**Figure 3:**
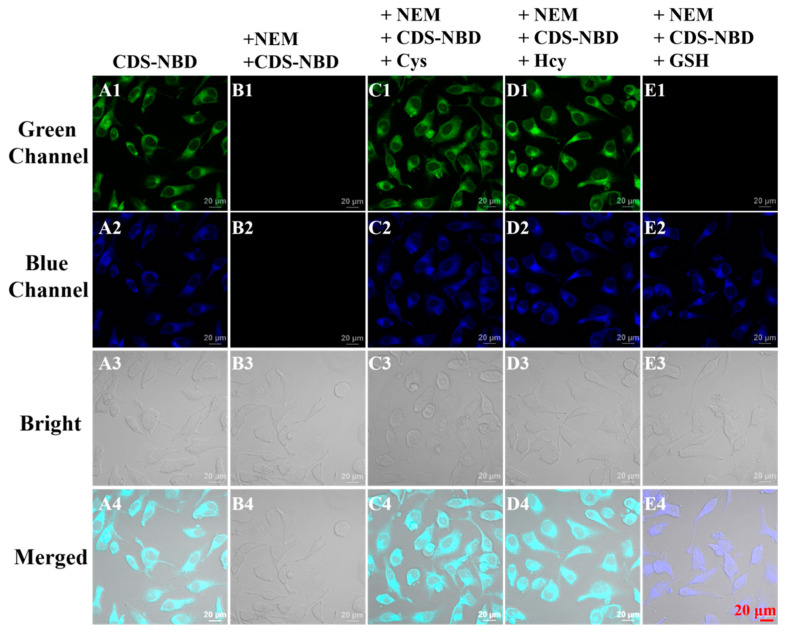
Confocal fluorescence images in HeLa cells. (**A1**–**A4**) cells incubated with probe **CDS-NBD** (10.0 μM). (**B1**–**B4**) cells pre-treated with NEM (1.0 mM) and incubated with probe **CDS-NBD** (10.0 μM), (**C**–**E**) cells pre-treated with NEM (1.0 mM), then successively incubated with probe **CDS-NBD** (10.0 μM) and different biothiols (60.0 μM for Cys (**C1**–**C4**), Hcy (**D1**–**D4**), and GSH (**E1**–**E4**), respectively). Blue channel (λ_ex_ = 405 nm, λ_em_ = 425–475 nm); Green channel (λ_ex_ = 488 nm, λ_em_ = 515–550 nm). Scale bar: 20 µm.

Using classical MTT assays, the cell cytotoxicity of probe **CDS-NBD** was first evaluated. As displayed in Figure S10, HeLa cells pre-incubated with 30.0 μM **CDS-NBD** for 24 h remained at over 93% of cell viability, indicating **CDS-NBD** had good biocompatibility and low cytotoxicity. Subsequently, to image biothiols in vitro, we attempted to choose HeLa cells as the test objects (Figure 3). As expected, **CDS-NBD** (10.0 μM)-loaded HeLa cells exhibited bright dual-fluorescence signals in blue (Figure 3A2) and green channels (Figure 3(A1)). In contrast, much weaker emission was observed from the above-mentioned channels (Figure 3(B1,B2)) after **CDS-NBD** (10.0 μM) was added to the NEM-treated HeLa cells (1.0 mM). These data supported that **CDS-NBD** could effectively image endogenous thiols in living HeLa cells. What is more, we also captured remarkable fluorescence signals in green (Figure 3C1,D1) and blue channels (Figure 3(C2,D2)) when HeLa cells pre-dealed with 1.0 mM N-ethylmaleimide (NEM, a typical biothiols depletion reagent, covalent sulfide bonds with sulfhydryls, enabling them to be permanently blocked to prevent disulfide bond formation) was successively stained with **CDS-NBD** (10.0 μM) and Cys/Hcy (100.0 μM). Further, HeLa cells pre-treated with N-ethylmaleimide (1.0 mM), **CDS-NBD** (10.0 μM), and GSH (100.0 μM) demonstrated only strong blue fluorescence emission (Figure 3(E1,E2)). Overall, **CDS-NBD** had good biocompatibility and could serve as an enabling tool for sensing biothiols in living cells by monitoring the changes of dual-fluorescence signals in blue and green channels.

### 2.7. Zebrafish Imaging 

**Figure 4:**
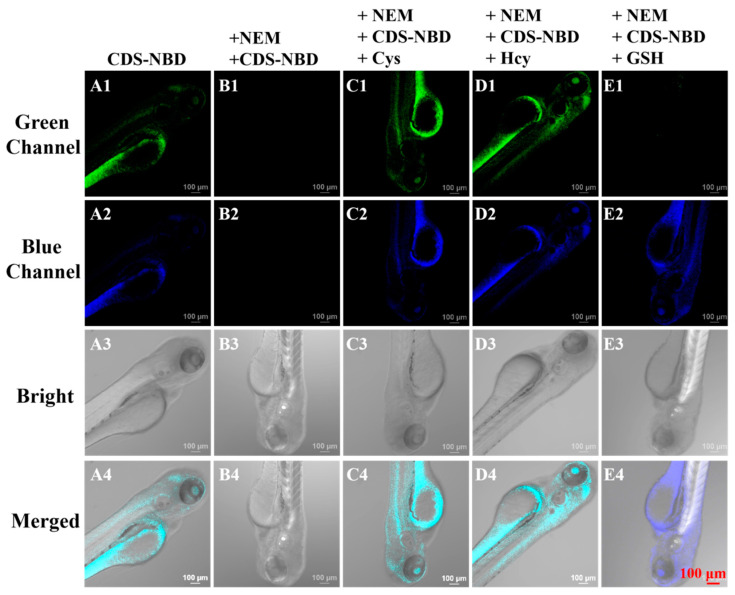
Confocal fluorescence images in zebrafish. (**A1**–**A4**) incubated with probe **CDS-NBD** (10.0 μM). (**B1**–**B4**) pre-treated with NEM (1.0 mM) and incubated with probe **CDS-NBD** (10.0 μM), (**C**–**E**) pre-treated with NEM (1.0 mM), then successively incubated with probe **CDS-NBD** (10.0 μM) and different biothiols ((60.0 μM for Cys (**C1**–**C4**), Hcy (**D1**–**D4**) and GSH (**E1**–**E4**), respectively). Blue channel (λ_ex_ = 405 nm, λ_em_ = 425–475 nm); Green channel (λ_ex_ = 488 nm, λ_em_ = 515–550 nm). Scale bar: 100 µm.

Motivated by the above results, using probe **CDS-NBD**, zebrafish imaging was next carried out on four-day-old zebrafish. As is shown in Figure 4(A1–A4), when zebrafish were incubated with **CDS-NBD** (10.0 μM), bright fluorescent signals in the blue and green channels could be found. By sharp contrast, when the zebrafish were cultured with 1.0 mM N-ethylmaleimide (NEM, free biothiol blocking reagent) and **CDS-NBD** (10.0 μM), negligible fluorescence both in the blue (Figure 4(B2)) and green channel (Figure 4(B1)) were detected. These phenomena demonstrated that probe **CDS-NBD** could be used to sense intracellular biothiols via dual-color fluorescence imaging. Additionally, after the treatment with N-ethylmaleimide (1.0 mM), **CDS-NBD** (10.0 μM), and Cys/Hcy (100.0 μM), the zebrafish sent out outstanding fluorescence in blue (Figure 4(C2,D2)) and green channels (Figure 4(C1,D1)). For GSH (100.0 μM), only an intense blue fluorescent signal (Figure 4(E1,E2)) in zebrafish was captured. Therefore, by employing two different fluorescence signals, probe **CDS-NBD** could monitor Cys/Hcy and GSH in vivo with outstanding performance. 

## 3. Experimental

### 3.1. Instruments and Reagents

Unless otherwise noted, all reagents and solvents were purchased from Chinese commercial suppliers and used for experiments without further purification. Nuclear magnetic resonance (NMR) spectra of probe **CDS-NBD** were collected using a Bruker Avance 600 MHz spectrometer. UV-vis absorption and fluorescence spectra through a Shimadzu UV-2450 spectrophotometer and HITACHI F-4600 fluorescence spectrophotometer. Fluorescence imaging was recorded using a Zeiss LSM710 Wetzlar (German) laser scanning confocal microscope. High-resolution mass spectra (HR-MS) data of synthesized new compounds were measured with AB Sciex TripleTOF 4600. 

### 3.2. General Procedure of Spectral Measurements

The parent aqueous solutions (10.0 mM) of various amino acids (Asp, Ala, Arg, His, Glu, Gln, Gly, Leu, Ile, Met, Lys, Phe, Pro, Tyr, Thr) and ions (K^+^, Zn^2+^, Mg^2+^, Na^+^, Ca^2+^, NO_3_^−^, CO_3_^2−^, NO_2_^−^, I^−^, SO_4_^2−^, SO_3_^2−^, F^−^, Cl^−^, Br^−^, ClO^−^) were prepared by the twice distilled water. The stock solution of probe **CDS-NBD** (1.0 mM) was dissolved in DMSO. The test solution of 3.0 mL quartz cuvette was obtained using 10.0 μM probe **CDS-NBD** mixed with an appropriate solution of analyte sample in PBS buffer (10 mM, pH = 7.4, 20% DMSO). The fluorescence spectroscopic data were acquired with the excitation wavelength at 355 nm/450 nm and 5.0/5.0 nm for slit width.

### 3.3. Cell Experiments

The HeLa cells were cultured and grown in DMEM containing 10% FBS with a humidified atmosphere of 5% CO_2_ overnight for cell attachment. The standard MTT method was applied to evaluate the cytotoxicity of probe **CDS-NBD** with disparate concentrations (0.0, 2.0, 5.0, 10.0, 15.0, 30.0 µM) for the viability of HeLa cells. For the cell imaging, the five groups of HeLa cells were handled as follows when HeLa cells were plated on culture dishes overnight to adhere. In the first group, HeLa cells were only incubated with probe **CDS-NBD** (10.0 μM) for 30 min. In the second group, as a control, HeLa cells were pre-interacted with thiol scavenger N-ethylmaleimide (NEM, 1.0 mM) for 30 min and then treated with the culture medium of probe **CDS-NBD** (10.0 μM) for another 30 min. Meanwhile, the other three experimental groups were pre-cultured with NEM and probe **CDS-NBD** solution and further incubated with (100.0 μM) different biothiols (Cys, Hcy, GSH). After rinsing with PBS buffer before imaging, confocal microscopy of HeLa cells was carried out on a Zeiss LSM 710.

### 3.4. Zebrafish Experiments 

The 4-day-old zebrafish were purchased from Eze-Rinka Company (Nanjing, China) and cultured in E3 medium on a light and dark cycle (13/11 h). Five experimental groups were established with different treatments, the same as cell imaging. In the first experimental group, zebrafish were only stained with probe **CDS-NBD** (10.0 μM) for 30 min. The remaining four experimental groups were all pre-treated with NEM (1.0 mM) at the same time. One of them, as a control, was incubated with probe **CDS-NBD** (10.0 μM) for another 30 min. Another three experimental groups were subsequently subjected to the addition of the probe **CDS-NBD** (10.0 μM) for 30 min and biothiols (100.0 μM for Cys/Hcy/GSH) for another 30 min. After removing the treatment liquid with PBS, confocal fluorescence imaging was acquired. 

### 3.5. Synthesis of Compound ***1***

**7-HCA** (44.0 mg, 0.2 mmol) and compound **2** (57.6 mg, 0.2 mmol) were dissolved in 18.0 mL CH_2_Cl_2_, EDCI (62.0 mg, 0.3 mmol), and DMAP (14.0 mg, 0.1 mmol) was then added. The mixture was stirred for 4 h at room temperature. The corresponding solids were purified by column chromatography on silica gel (CH_2_Cl_2_: MeOH = 20:1) to obtain compound **1** (59 mg, 60.2%). ^1^H NMR (600 MHz, DMSO) δ 10.72 (s, 1H), 10.61 (s, 1H), 8.48 (d, *J* = 7.2 Hz, 1H), 7.86 (d, *J* = 7.0 Hz, 2H), 7.69 (dd, *J* = 20.5, 7.7 Hz, 3H), 6.96–6.70 (m, 3H), 6.28 (s, 1H), 3.98 (s, 2H).^13^C NMR (151 MHz, DMSO) δ 167.80, 161.76, 160.65, 155.52, 151.08, 148.91, 143.31, 141.98, 141.32, 136.93, 133.21, 133.00, 130.12, 127.23, 123.43, 121.34, 119.50, 113.52, 112.54, 111.93, 102.85. HRMS (EI) m/z calcd for [C_23_H_14_N_4_O_7_S+H]^+^: 491.0661, Found: 491.0638. 

### 3.6. Synthesis of Probe ***CDS-NBD***

Compound **1** (49.0 mg, 0.1 mmol) and triethylamine (13.2 mg, 0.13 mmol), as well as 2,4-dinitrobenzenesufonyl chloride (35.1 mg, 0.13 mmol), were mixed in 15.0 mL CH_2_Cl_2._ After stirring at room temperature for 4 h, the resulting solution was extracted three times with CH_2_Cl_2_ and washed to neutrality with saturated salt water. The combined organic layers were subjected to concentrate under reduced pressure and purified on silica gel chromatography with CH_2_Cl_2_/EtOAc (20:1) as eluent to obtain a faint yellow powder (51.9 mg, 72.1%). ^1^H NMR (600 MHz, DMSO) δ 10.74 (s, 1H), 9.13 (d, *J* = 2.1 Hz, 1H), 8.63 (dd, *J* = 8.7, 2.2 Hz, 1H), 8.49 (d, *J* = 8.0 Hz, 1H), 8.35 (d, *J* = 8.7 Hz, 1H), 7.92 (d, *J* = 8.8 Hz, 1H), 7.84 (d, *J* = 8.6 Hz, 2H), 7.70 (d, *J* = 8.6 Hz, 2H), 7.40 (d, *J* = 2.3 Hz, 1H), 7.30 (dd, *J* = 8.8, 2.3 Hz, 1H), 6.79 (d, *J* = 8.0 Hz, 1H), 6.63 (s, 1H), 4.08 (s, 2H). ^13^C NMR (151 MHz, DMSO) δ 167.51, 159.49, 154.15, 152.11, 150.48, 150.05, 148.91, 148.60, 143.31, 141.87, 141.27, 136.91, 134.06, 133.23, 132.99, 132.00, 131.16, 129.13, 128.19, 123.42, 121.79, 121.34, 119.59, 119.55, 118.81, 117.62, 111.09. HRMS (EI) m/z calcd for [C_29_H_17_N_6_O_13_S_2_+H]^+^: 721.0295, Found: 721.0256. 

## 4. Conclusions

In summary, we constructed a novel fluorescent probe **CDS-NBD** based on **7-HCA** for the detection of Cys/Hcy and GSH through two different emission channels by using DNBS and NBD as the highly sensitive and selective dual response sites for thiols. **CDS-NBD** had **a** large separation of the emission wavelengths, fast response, notable fluorescence enhancement, and excellent sensitivity and selectivity. Importantly, probe **CDS-NBD** could make a distinction between GSH (blue fluorescence) and Cys/Hcy (mixed blue-green fluorescence) by two different fluorescent signals. Furthermore, dual-color fluorescence imaging experiments in living HeLa cells and zebrafish also demonstrated that **CDS-NBD** could serve as a useful tool for the detection of Cys/Hcy and GSH. 

## Data Availability

All data generated or analyzed during this study are included in this published article (and its Supplementary Information files) or are available from the corresponding author upon reasonable request.

## Supplementary Materials

The following supporting information can be downloaded at: https://www.mdpi.com/article/10.3390/bios12111056/s1, Table S1: The reported fluorescent probes based on dual-site for thiols, Figure S1: UV-vis absorption spectra of probe **CDS-NBD** (black) and reacted with Cys (red), Hcy (green) and GSH (blue) in PBS buffer, Figure S2: UV-vis absorption (black) and fluorescence (blue) spectra of **7-HCA** in PBS buffer, Figure S3: UV-vis absorption (black) and fluorescence (green) spectra of NBD-N-Bu in PBS buffer, Figure S4: The change of fluorescence intensity of the probe **CDS-NBD** in PBS with the addition of 100.00 μM biologically relevant ions at 470 nm of emission, Figure S5: The change of fluorescence intensity of the probe **CDS-NBD** in PBS with the addition of 100.00 μM biologically relevant ions at 557 nm of emission, Figure S6: Fluorescence responses of probe **CDS-NBD** toward Cys in the present of various coexistence substances (100.00 μM) at 470 nm of emission, Figure S7: Fluorescence responses of probe **CDS-NBD** toward Cys in the present of various coexistence substances (100.00 μM) at 557 nm of emission, Figure S8: pH effect on the fluorescence intensity of probe **CDS-NBD** (10.0 μM) without (blue) and with (60.0 μM) biothiols (red: Cys, green: Hcy, blue: GSH) at 470 nm, Figure S9: pH effect on the fluorescence intensity of probe **CDS-NBD** (10.0 μM) without (blue) and with (60.0 μM) biothiols (red: Cys, green: Hcy, blue: GSH) at 557 nm, Figure S10: Percentage of viable HeLa cells after treatment with indicated concentrations of probe **CDS-NBD** after 24 h, Figure S11: ^1^H NMR spectrum of compound **1** in DMSO-*d*_6_, Figure S12: ^13^C NMR spectrum of compound **1** in DMSO-*d*_6_, Figure S13: Mass spectrum of compound **1**, Figure S14: ^1^H NMR spectrum of probe **CDS-NBD** in DMSO-*d*_6_, Figure S15: ^13^C NMR spectrum of probe **CDS-NBD** in DMSO-*d*_6_, Figure S16: Mass spectrum of probe **CDS-NBD**, Figure S17: Mass spectrum of probe **CDS-NBD** with GSH, Figure S18: Mass spectrum of probe **CDS-NBD** with Cys.
