# Supplementary material for: Dual Response Site Fluorescent Probe for Highly Sensitive Detection of Cys/Hcy and GSH In Vivo through Two Different Emission Channels
Source: Biosensors (Basel). 2022 Nov 21;12(11):1056. doi: 10.3390/bios12111056 (PMC9688468; doi:10.3390/bios12111056)
Supplement: Supplementary file 1 [file biosensors-12-01056-s001.zip › biosensors-2006284-supplementary.pdf]

Supporting information for

Dual response site fluorescent probe for highly sensitive detection of Cys/Hcy and GSH in vivo through two different emission channels

Huiling Hou [a], Qi Liu [b], Xiangbao Liu [c], Shuang Fu [c], Hongguang Zhang [c], Shuang Li [c], Song Chen [c], Peng Hou [c]*

[a]Achievement Transformation Center, Qiqihar Medical University, Qiqihar, P. R. China, 161006.

[b]Research Institute of Medicine & Pharmacy, Qiqihar Medical University, Qiqihar, P. R. China, 161006.

[c]College of Pharmacy, Qiqihar Medical University, Qiqihar, P. R. China, 161006.

* *Corresponding author.*

E-mail address: houpeng1982@163.com

Table of contents

Page

Table S1 .... **1**

Figures S1-2 .... **3**

Figures S3-4 .... **4**

Figures S5-6 .... **5**

Figures S7-8 .... **6**

Figures S9-10 .... **7**

Figures S11-12 .... **8**

Figures S13-14 .... **9**

Figures S15-16 .... **10**

Figures S17-18 .... **11**

Table S1. The reported fluorescent probes based on dual-site for thiols.

Probe	Stokes shift	Test system	Detection limit	Response time	Application	Literature
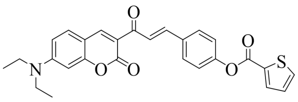	51nm	HEPES: DMSO= 6:4	Cys:0.24μM	Cys:10min	HeLa cells BHK-21 cells	Dyes Pigments 2022, 197, 109823
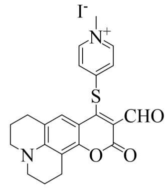	104nm/ 61nm	PBS buffer	Cys: 132nM Hcy: 105nM GSH :62nM	Cys: 190s Hcy: 155s GSH: 80s	zebrafish embryos; HeLa cells	Dyes Pigments 2022, 199, 110058
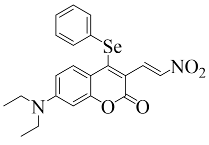	Cys: 95nm Hcy: 92nm GSH: 62nm	PBS: DMSO= 9:1	Cys: 39.2nM Hcy: 126.7nM GSH: 839.5nM	Cys: 12min Hcy: 12min GSH: 20min	HepG2 cells	Dyes Pigments 2022, 203, 110312
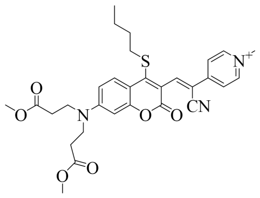	Cys: 96nm Hcy: 97nm GSH: 90nm	PBS: DMSO= 8:2	Cys: 27.3nM Hcy: 45.9nM GSH: 13.7nM	-------	HepG2 cells ; zebrafish	Chinese Chem. Lett. 2022, 33, 1609-1612
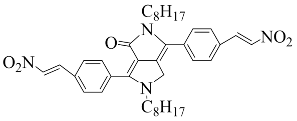	59nm	PBS: THF= 2:3	6.14×10^{-8}M	Cys: 1min Hcy: 3min GSH: 16min	HeLa cells	Sensor Actua B-Chem. 2017, 244, 531-540
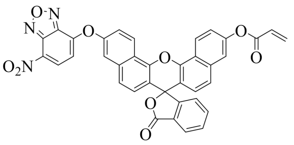	Cys: 87nm Hcy: 73nm	PBS with 1.0 mM CTAB	Cys: 1.5×10^{-7}M Hcy: 1.1×10^{-7}M	60min	HeLa cells	Tetrahedron Lett. 2018, 59, 2232-2237
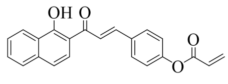	140nm	PBS: DMSO= 1:1	Cys： 9.1×10^{-8}M	Cys:10min	U87 cells	Biosens. Bioelectron 2017, 92, 583-588

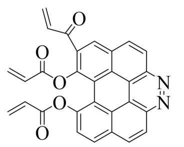	65nm	PBS buffer	0.11μM	5min	HeLa cells	Sensor Actua B-Chem. 2020, 320, 128304
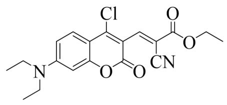	Hcy: 78nm Cys: 80nm GSH: 81nm	PBS: DMSO= 7:3	Hcy: 3nM, Cys:6nM GSH: 200nM	15min	A375 cells	Talanta 2020, 219, 121353
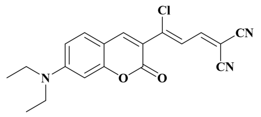	60nm	PBS: DMSO= 1:1	0.3μM	7min	A549 cell	Sensor Actua B-Chem .2 017, 253, 42-49
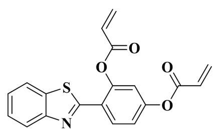	113nm	HEPES: DMSO= 9:1	0.8μM	-------	HeLa cells	Dyes Pigments 2017, 139, 73-78
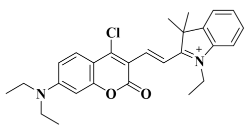	76nm	CH_3CN: HEPES= 1:9	4.63×10^{-7}M	------	Hi5 cell C. eleg-an s	Chinese Chem. Lett. 2017, 28, 2023-2026
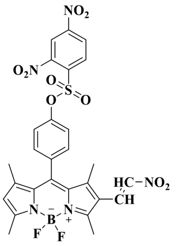	-------	CH_3CN: HEPES= 1:1	Hcy: 87nM Cys: 147nM GSH: 129nM	Cys: 6.7min Hcy: 13.5min GSH: 79.4min	HeLa cells	Dyes Pigments 2018, 152, 29-35
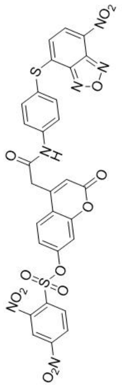	**Cys/ Hcy/GS H: 117 nm**	**PBS: DMSO= 8:2**	**Cys: 32nM Hcy: 45nM GSH :22nM**	**Cys: 300s Hcy: 350s GSH: 350s**	**HeLa cells Zebrafish**	**This work**

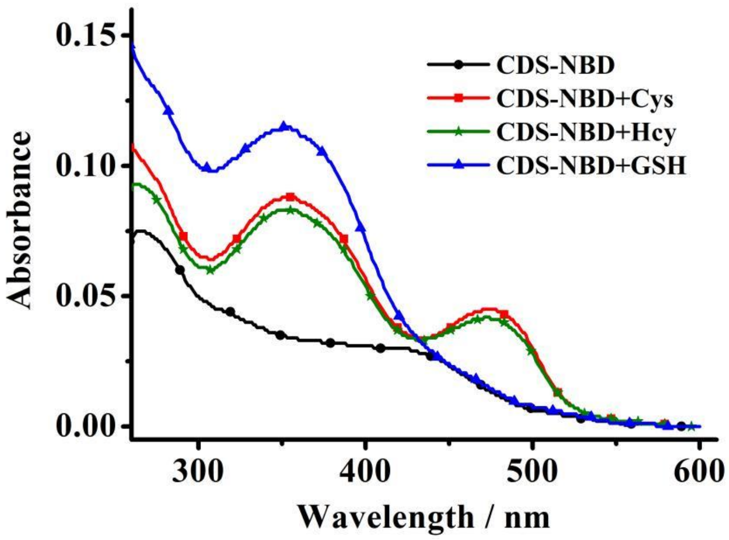

Figure S1 UV-vis absorption spectra of probe **CDS-NBD** (black) and reacted with Cys (red), Hcy (green) and GSH (blue) in PBS buffer.

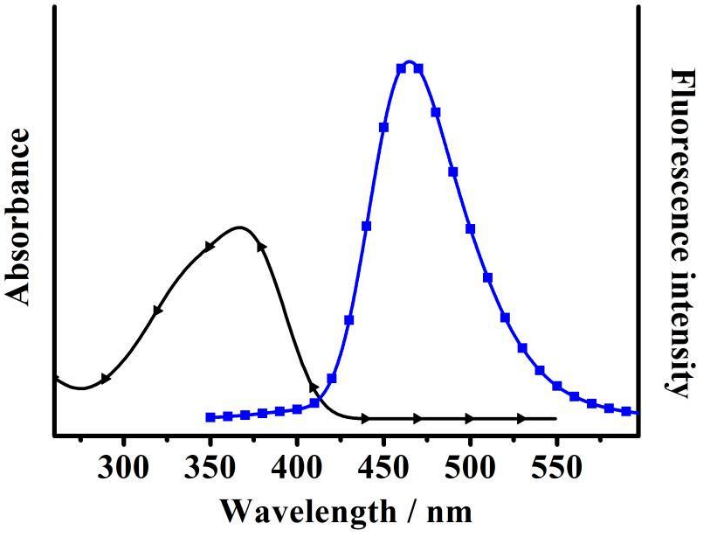

Figure S2 UV-vis absorption (black) and fluorescence (blue) spectra of **7-HCA** in PBS buffer.

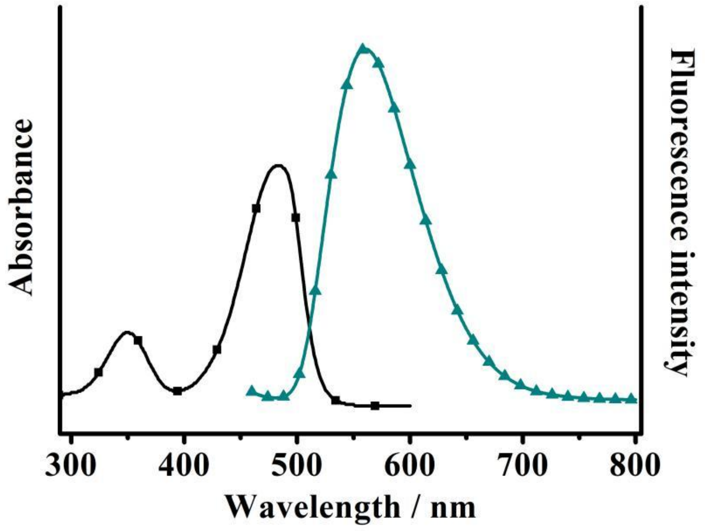

Figure S3 UV-vis absorption (black) and fluorescence (green) spectra of **NBD-N-Bu** in PBS buffer.

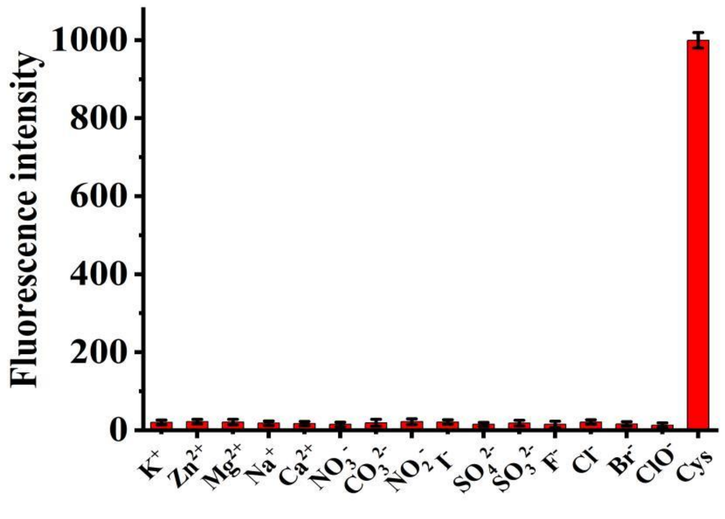

Figure S4 The change of fluorescence intensity of the probe **CDS-NBD** in PBS with the addition of 100.00 μM biologically relevant ions at 470 nm of emission.

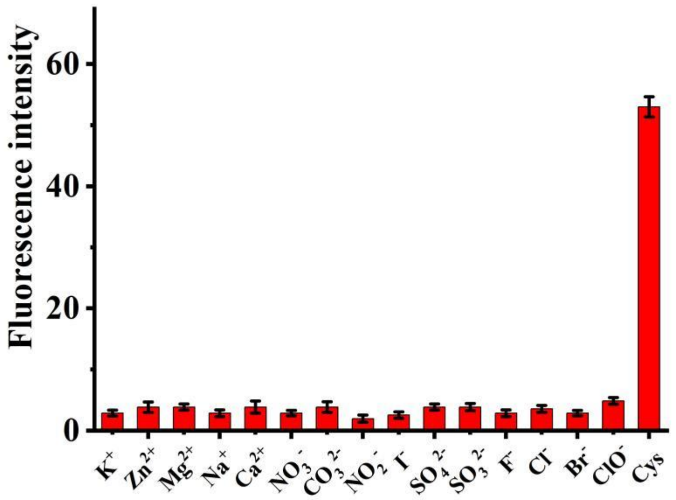

Figure S5 The change of fluorescence intensity of the probe **CDS-NBD** in PBS with the addition of 100.00 μM biologically relevant ions at 557 nm of emission.

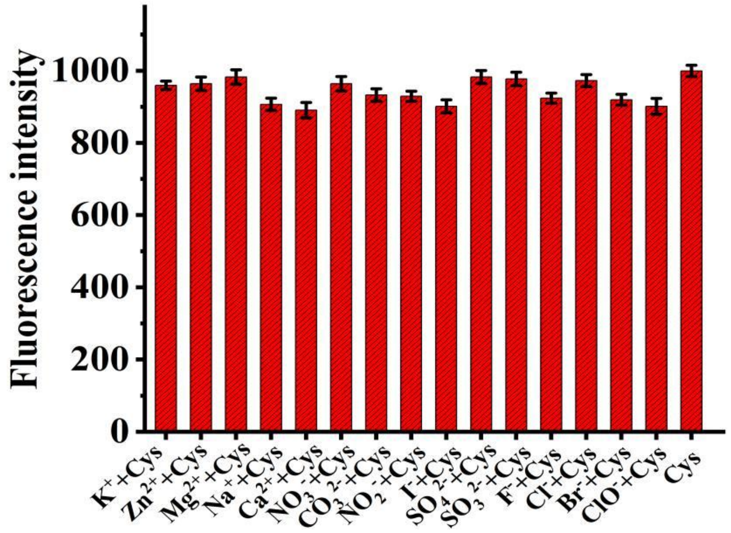

Figure S6 Fluorescence responses of probe **CDS-NBD** toward Cys in the present of various coexistence substances (100.00 μM) at 470 nm of emission.

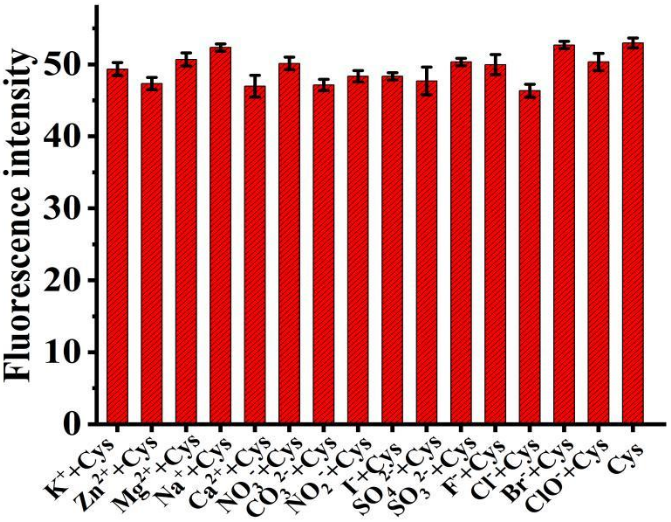

Figure S7 Fluorescence responses of probe **CDS-NBD** toward Cys in the present of various coexistence substances (100.00 μM) at 557 nm of emission.

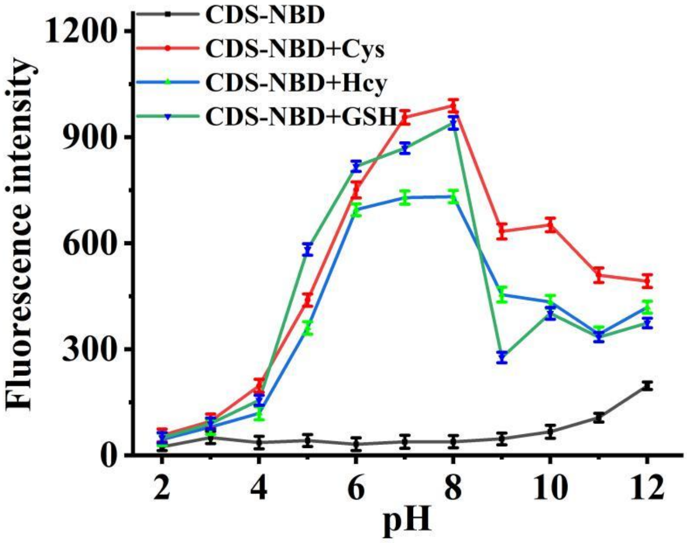

Figure S8 pH effect on the fluorescence intensity of probe **CDS-NBD** (10.0 μM) without (blue) and with (60.0 μM) biothiols (red: Cys, green: Hcy, blue: GSH) at 470 nm.

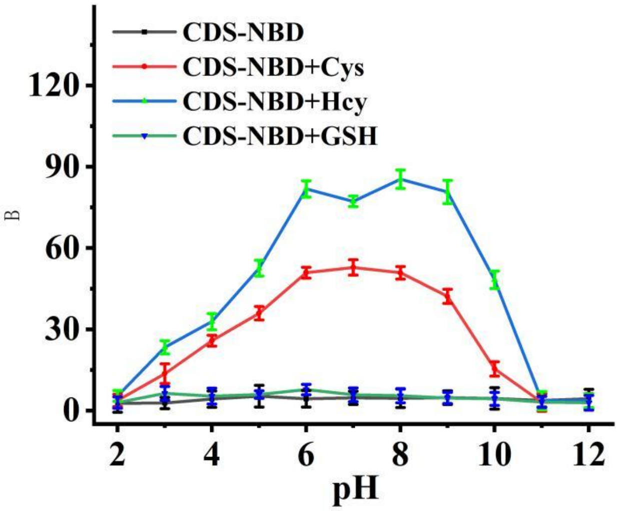

Figure S9 pH effect on the fluorescence intensity of probe **CDS-NBD** (10.0 μM) without (blue) and with (60.0 μM) biothiols (red: Cys, green: Hcy, blue: GSH) at 557 nm.

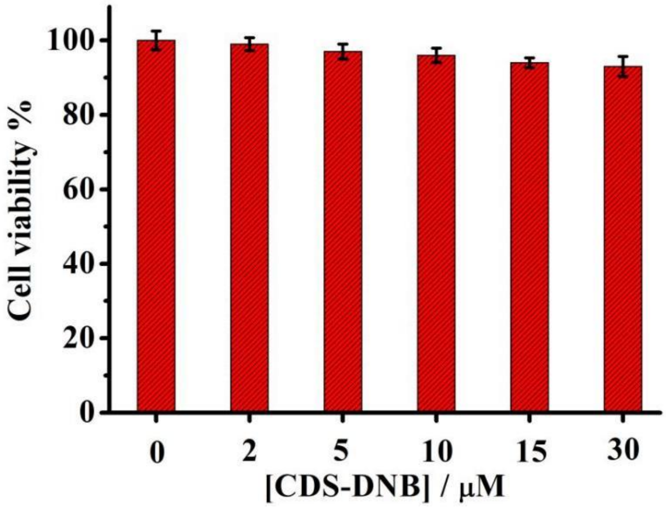

Figure S10 Percentage of viable HeLa cells after treatment with indicated concentrations of probe **CDS-NBD** after 24 hours.

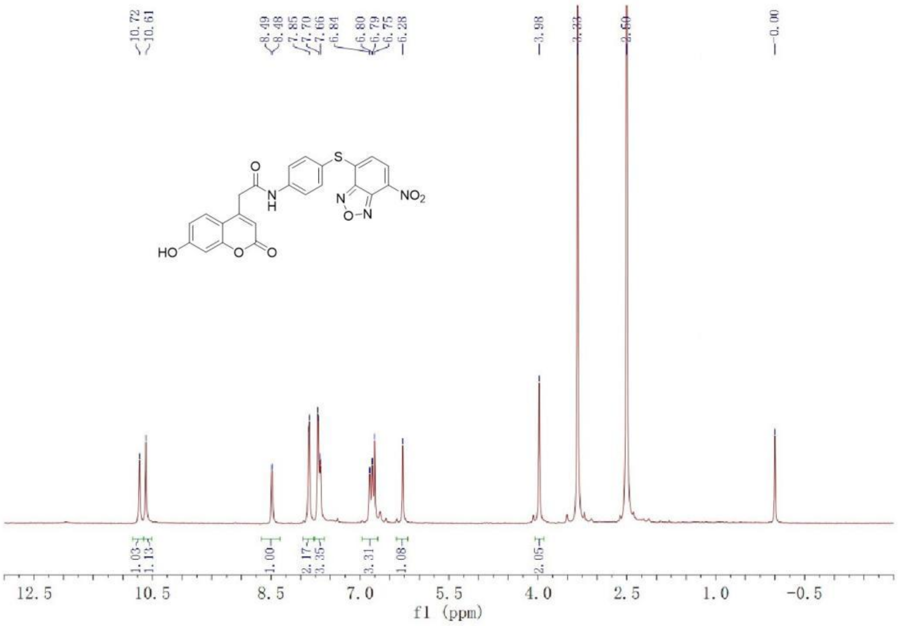

Figure S11 ^{1}H NMR spectrum of compound **1** in DMSO-d_6.

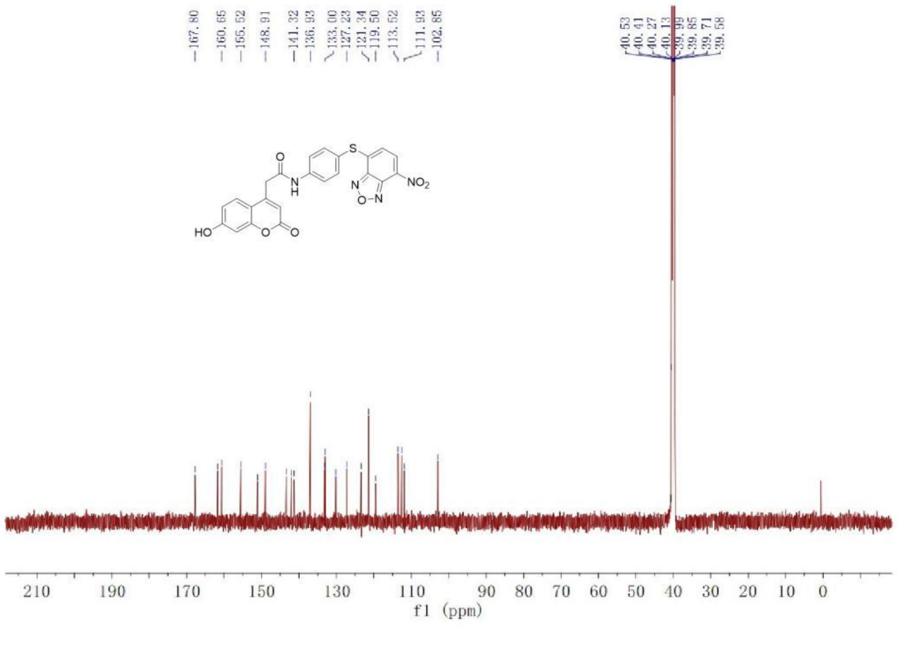

Figure S12 ^{13}C NMR spectrum of compound **1** in DMSO-d_6.

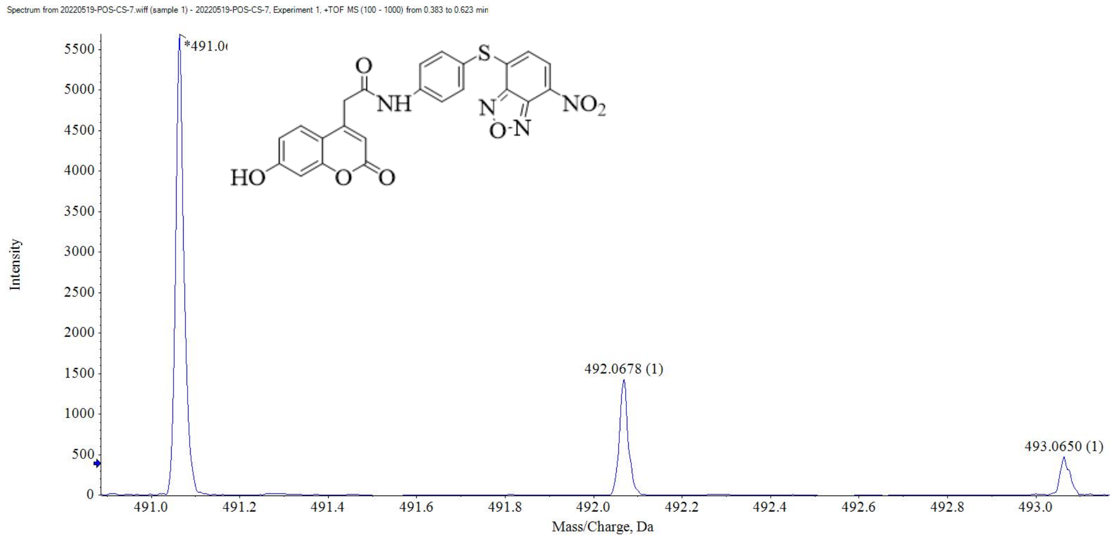

Figure S13 Mass spectrum of compound **1**.

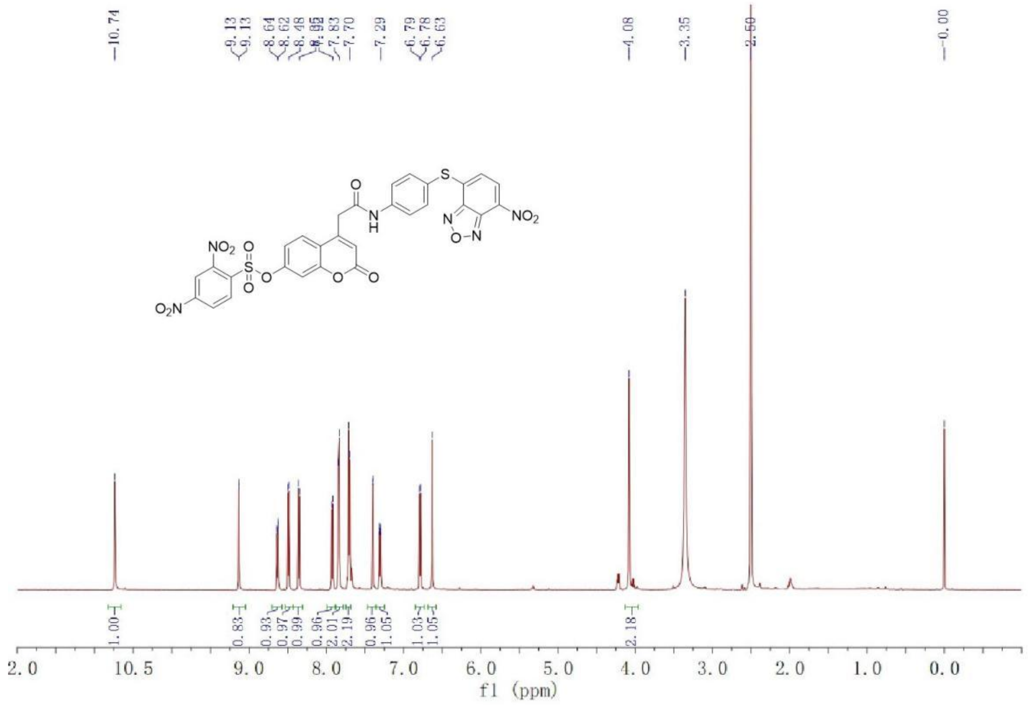

Figure S14 ^{1}H NMR spectrum of probe **CDS-NBD** in DMSO-d_6.

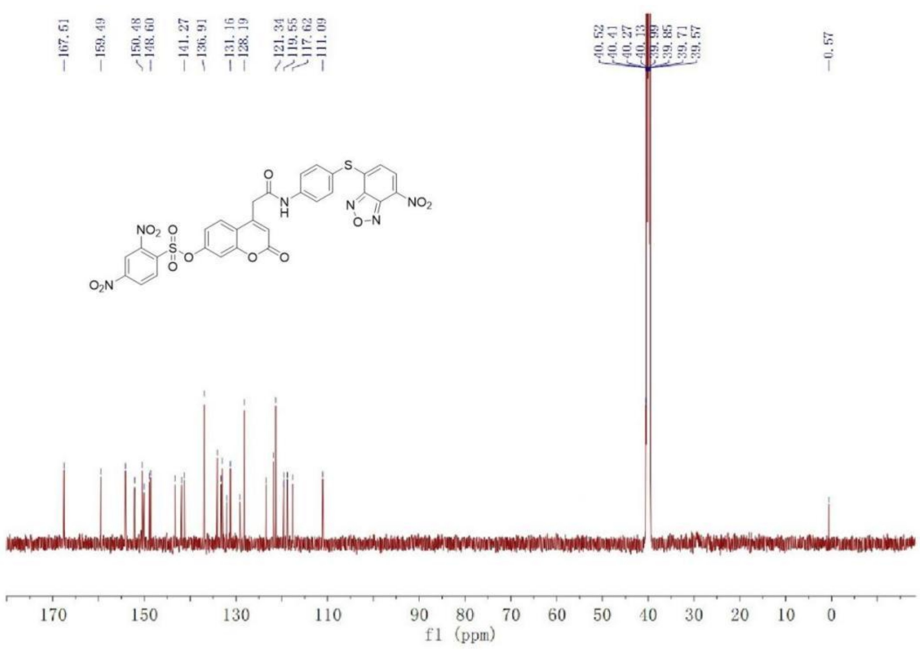

Figure S15 ^{13}C NMR spectrum of probe **CDS-NBD** in DMSO-d_6.

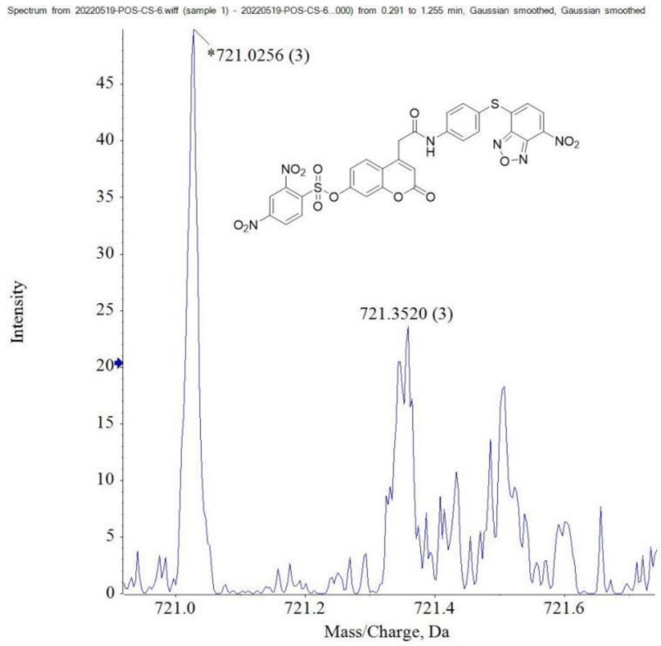

Figure S16 Mass spectrum of probe **CDS-NBD**.

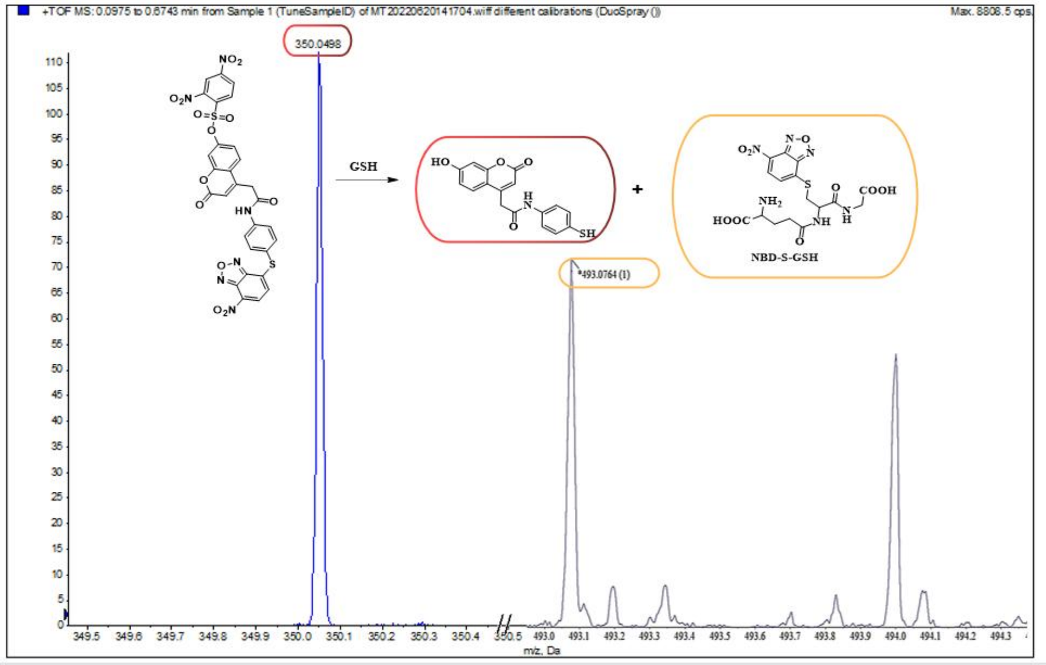

Figure S17 Mass spectrum of probe **CDS-NBD** with GSH.

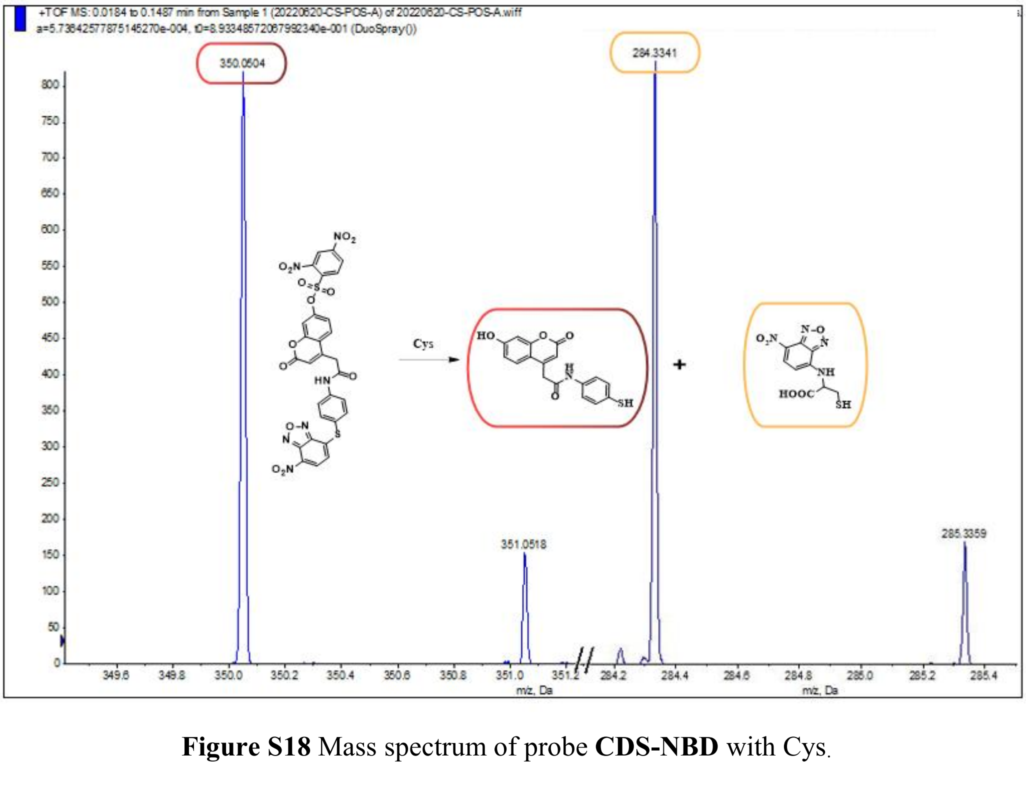

Figure S18 Mass spectrum of probe **CDS-NBD** with Cys.
